# Novel AlphaPlex Design Enables Rapid Differentiation of *Campylobacter* Species

**DOI:** 10.3390/pathogens15090884

**Published:** 2026-08-24

**Authors:** Cheryl M. Armstrong, Sarah Nguyen, Yiping He, Manita Guragain

**Affiliations:** Eastern Regional Research Center, United States Department of Agriculture, Agricultural Research Service, Wyndmoor, PA 19038, USAyiping.he@usda.gov (Y.H.); manita.guragain@usda.gov (M.G.)

**Keywords:** *Campylobacter*, oligo-Alpha, multiplex detection, ELISA, pathogen

## Abstract

*Campylobacter jejuni* and *Campylobacter coli* are major foodborne pathogens whose accurate species-level discrimination is important for outbreak investigations as well as rapidly assessing putative antimicrobial resistance profiles and the pathogenic potential of the bacterium. To facilitate species differentiation, a novel assay that integrates the nucleic acid-sensing capability of the oligo-Alpha with the multiplexing capacity of the AlphaPlex bead chemistries was developed. This wash-free system (designated as oligo-Plex) enables the detection and differentiation of *C. jejuni* and *C. coli* within a single reaction and can be completed in approximately 75 min. It works by using custom oligonucleotides modified for bead attachment, which hybridize sequentially along *Campylobacter*’s *glyA* gene and ultimately bridge the donor and acceptor beads. Improvements in assay stringency were made by increasing incubation temperatures, thus allowing the resolution of target from non-target. Comparisons of FITC–europium and DIG–terbium labeling systems revealed superior performance by the FITC–europium pair and suggested that helical positioning and steric accessibility likely influence donor–acceptor efficiency. Maximized signal separation was seen when using terbium for the detection of *C. coli* and europium for the detection of *C. jejuni*. Testing was performed in a Tris-based buffer and milk to confirm matrix tolerance, with potential areas for further optimization identified. The oligo-Plex presented here establishes a streamlined, adaptable platform suitable for high-throughput screening of multiple nucleic acid analytes that is readily extendable to a diverse array of pathogens through appropriate oligo selection.

## 1. Introduction

The *Campylobacter* spp. are Gram-negative, microaerophilic bacteria known primarily for their role in foodborne illness worldwide, with clinical outcomes that can include diarrhea, bacteremia, and sequelae such as Guillain–Barré syndrome [1]. Although more than 20 species of *Campylobacter* have been described, not all are associated with human illness [2]. The two most notable species causing human illness are *C. jejuni* and *C. coli*, with the U.S. Centers for Disease Control and Prevention reporting *C. jejuni* as the responsible agent for almost 90% of human *Campylobacter* illnesses [3]. *Campylobacter* spp. are frequently found in the intestines of animals used as food sources, such as sheep, cattle, and poultry, with infection occurring when undercooked or cross-contaminated foods are consumed [4]. *Campylobacter* can also be found in milk and milk products, with outbreaks commonly associated with raw/unpasteurized milk [5]. Given the high prevalence of *Campylobacter* on foods and its diverse array of animal and environmental reservoirs, accurate species identification can aid with traceback during outbreak investigations. Because different species can have distinct host associations, speciation can help pinpoint outbreak sources while aiding with epidemiological surveillance. For example, *C. coli* is more common in swine, *C. jejuni* in poultry, *C. fetus* in cattle and sheep, and *C. lari* in shellfish [6]. Variations in survival characteristics and antimicrobial resistance profiles have also been found amongst the different species [7,8,9], which can affect public health interventions, including reservoir-specific interventions, and clinical management based upon their antimicrobial resistance profiles. This is especially important as the number of multidrug-resistant strains isolated from animals rises [7]. Therefore, techniques that can make such distinctions are key to maintaining the balance between mitigating the burden of foodborne pathogens and avoiding unnecessary waste through the removal of foods that do not pose a risk to human health.

Various techniques are available that enable accurate species-level identification based upon genetic differences. The most common include amplification of specific genes via polymerase chain reaction (PCR) and the employment of genome sequencing. Unfortunately, PCR-based techniques can be sensitive to inhibitors naturally present in food matrices, while genome sequencing is substantially more expensive and time-intensive, due in part to the expertise in bioinformatics required for its implementation [10,11,12]. Given the ubiquitous nature of DNA, additional diagnostics that rapidly identify minor differences within genome sequences would be beneficial not only for ensuring the safety of our food supply but in other fields of study as well.

Modified approaches to the traditional enzyme-linked immunosorbent assays (ELISAs), such as ELISA-based DNA hybridization or PCR-ELISA, have been used to identify the presence of foodborne pathogens via genetic determinants [13,14]. However, these methods are also susceptible to inhibitors commonly found in foods (e.g., ascorbic acid and p-Aminobenzoic acid) due to their reliance on the activity of enzymes such as horseradish peroxidase [15,16] and are therefore not widespread for food-safety diagnostics. A more recent offshoot of the ELISA is the amplified luminescent proximity homogenous assay-linked immunosorbent assay (AlphaLISA). AlphaLISAs are bead-based assays that employ specific wavelengths of light (typically within the ultraviolet range) to excite donor beads coated with photosensitizers, thus triggering the generation of singlet oxygen molecules. The singlet oxygen molecules then interact with acceptor beads located in the vicinity, which have been brought into close proximity through the binding of a common target. This cascade of events causes the acceptor bead to ultimately emit a distinct wavelength of light, creating a unique, measurable spectral signature [17]. AlphaLISAs are not only amenable to high-throughput applications, since they eliminate the wash steps from traditional ELISA protocols, but are also very robust due to their independence from enzymes, which makes them well suited for food-safety applications.

Although widely used as a tool for quantifying targets based upon protein–protein or DNA–protein interactions, our previous work has explored the application of the amplified luminescent proximity homogenous assay for nucleic acid-based interactions [18]. The assay, aptly named “oligo-Alpha”, employed oligonucleotides to detect a single DNA target. Interestingly, the commercial availability of several different acceptor beads with unique spectral signatures allows for the development of diagnostics that can simultaneously quantify multiple targets within a single reaction well while minimizing potential crosstalk. Assays employing this multiplexing technique have been coined “AlphaPlex” assays. Considering that simultaneous detection of multiple targets is highly relevant for food-safety diagnostics, since pathogens can co-occur within samples [19,20], application of this type of assay appears appropriate. However, published examples where ELISA-based DNA hybridization, PCR-ELISA, or the oligo-Alpha were used for the simultaneous detection of multiple foodborne pathogens in a single reaction were not found.

Presented here is an AlphaPlex assay designed for the simultaneous detection and differentiation of *C. jejuni* and *C. coli*. This assay combines the sophisticated energy transfer mechanism between donor and acceptor beads typical for AlphaPlex with the nucleic-acid sensing ability of the oligo-Alpha to enable highly sensitive multi-target detection of these foodborne pathogens. Benefits include a simple 96-well plate format where reagents are added sequentially into the well without the need for tedious wash steps and can be performed in approximately 75 min. Although this assay was designed to detect *C. jejuni* and *C. coli*, the method serves as a template for the detection of other nucleic acid targets of interest since it represents the first use of the AlphaPlex platform for DNA target differentiation.

## 2. Materials and Methods

### 2.1. Oligonucleotide Design and AlphaPlex Bead Selection

**Figure 1:**
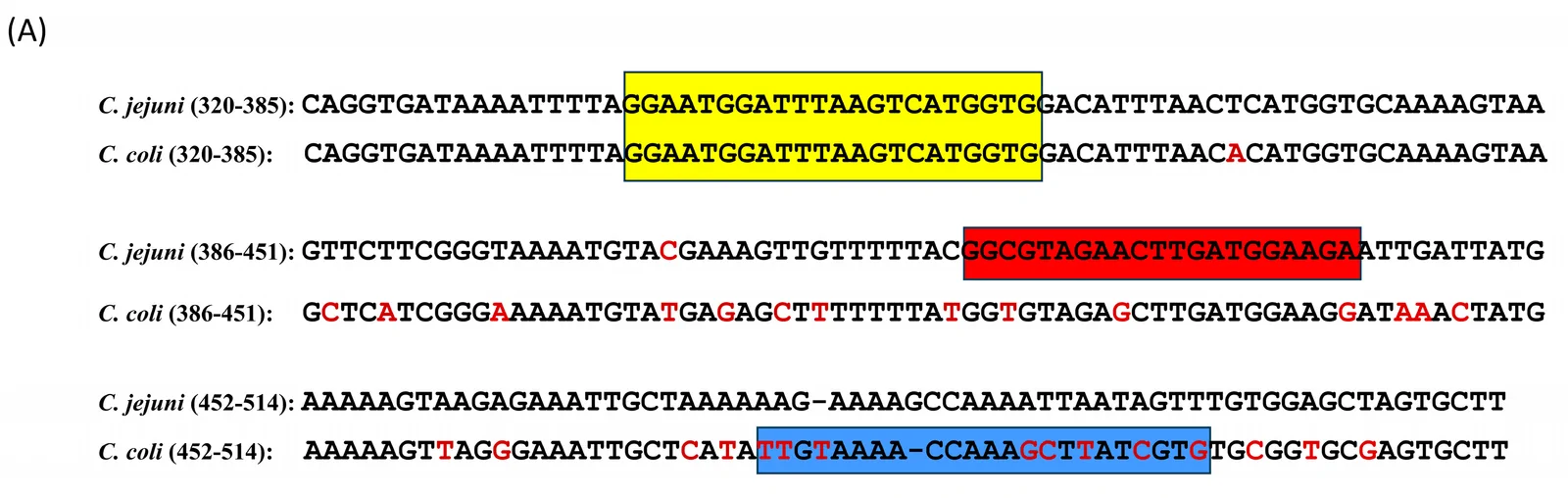

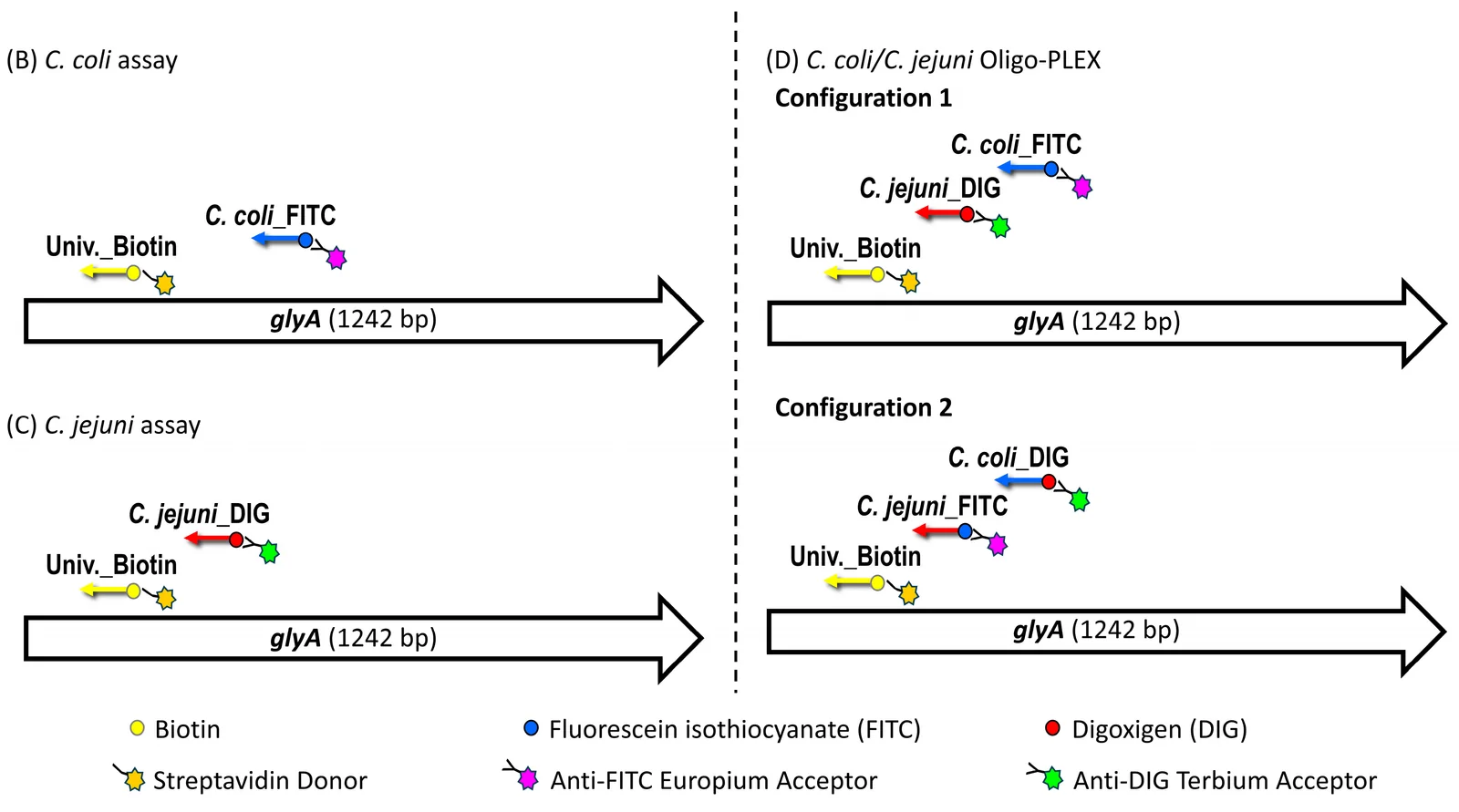
Design of oligo-Plex assay for *Campylobacter* detection. (**A**) Nucleotide alignment of the *glyA* region from *C. jejuni* and *C. coli* with insertions (dashes) and single nucleotide polymorphisms (red letters) indicated. The locations of the acceptor-binding oligos used for the detection of *C. jejuni* (highlighted red) and *C. coli* (highlighted blue) are noted, with the universal donor-binding oligo designated by the yellow-highlighted area. (**B**) Oligo-Alpha assays specific to *C. coli* with oligos (colored arrows) and their modifications, which allow for their conjugation to specific beads, are shown as colored circles. Streptavidin-coated donor beads are designated by orange stars, and the anti-FITC-coated europium acceptor beads are shown as purple stars. (**C**) Oligo-Alpha assays specific to *C. jejuni* with oligos (colored arrows) and their modifications are shown as colored circles. Streptavidin-coated donor beads are designated by orange stars, and the anti-DIG-coated terbium acceptor beads are green stars. (**D**) Oligo-Plex assays for *C. coli* and *C. jejuni* with oligos (colored arrows), their modifications (colored circles), and donor beads/acceptor beads (colored stars). Note the interchange of oligo modifications and acceptor beads between assay 1 and assay 2.

Oligonucleotides containing sequences representative of the *glyA* gene (Supplemental Table S1) were synthesized by either Bio-Synthesis Inc. (Bio-Synthesis Inc., Lewisville, TX, USA) or Integrated DNA Technologies, Inc. (IDT, Inc., Coralville, IA, USA). This region was chosen for its ability to differentiate *C. coli* from *C. jejuni*, as previously reported [21]. The *C. coli*-specific oligo, C. coli_FITC, was modified from primer CCF [22] to contain two extra nucleotides at the 3′ end, was made to the antisense strand of DNA compared to primer CCF, and contained the fluorescein isothiocyanate (FITC) modification at its 5′ end. These modifications were necessary to fit within the temperature parameters of the current assay and the technical requirements to allow conjugation to the AlphaLISA anti-FITC Acceptor beads (Cat # AL127C) (Revvity, Inc., Waltham, MA, USA). Oligo Campy_Bio was then designed based upon sequence alignments to an upstream region within *glyA* that was identical in both *C. coli* and *C. jejuni*, whereas the *C. jejuni*-specific oligo, C. jejuni_DIG, targets sequences unique to *C. jejuni* (Figure 1). Modifications, which included the addition of a dual biotin or digoxyigenin (DIG) label, supported their ability to bind to the Alphascreen Streptavidin Donor beads (Cat # 6760002) and the custom-made AlphaPlex 545 anti-DIG Terbium (Tb) acceptor beads (Cat # CUSM04964000EA) produced by Revvity, Inc. (Revvity, Inc., Waltham, MA, USA), respectively.

### 2.2. Oligo-Plex Assay Format

Assays were performed in 96-well ½ area gray plates (Revvity, Inc. Cat #6002350), with TopSeal-A film (Cat #6050185) (Revvity, Inc., Waltham, MA, USA) being used to seal the assay plates. The 1X assay buffer containing 10 mM Tris-Cl, 4 mM MgCl_2_, 50 mM KCl, and 200 µg/mL bovine serum albumin at pH 8.0 (TKMB buffer) previously reported for use with the oligo-Alpha was employed for the dilution of all assay reagents [18]. Stock solutions (500 nM) of all oligos were further diluted as reported in subsequent sections below. Single-stranded DNA representing a fragment of the *glyA* gene from either *C. coli* or *C. jejuni* was also synthesized for use in testing (IDT, Inc., Coralville, IA, USA). Assay conditions were similar to those previously reported except that assays were performed at 54 °C unless otherwise stated [18]. Signals were read using the Envision Multilabel plate reader (Perkin Elmer, Shelton, CT, USA) using the following conditions: (1) The temperature control was set at 54 °C (unless otherwise noted) for the duration of the experiment. (2) Shaking was performed for 30 s at 60 RPM with a 10 mm-diameter inside orbital shake. (3) Plates were incubated for 30 min. Signals were read via the AlphaScreen mirror with either the (4) Terbium (Tb) emission filter (545 nm) with an excitation time of 0.18 s and a total measurement time of 0.55 s or the (5) Europium (Eu) emission filter (615 nm) with an excitation time of 0.18 s and a total measurement time of 0.55 s. A general overview diagramming the steps of oligo-Plex is provided (Supplemental Figure S1), with specific details regarding individual assays listed below. All steps containing the AlphaPlex beads were performed under limited light conditions to avoid premature photosensitizer activation and photobleaching. Negative controls were added in the form of competing DNA (*C. coli* DNA in the *C. jejuni* assay and *C. jejuni* DNA in the *C. coli* assay), while sterile water was used for the no-DNA negative control throughout the study.

### 2.3. Optimization of Universal and Species-Specific Oligo Concentrations

Optimal concentrations of oligos used for detecting *C. coli* were empirically derived by varying the amount of Campy_Bio and C. coli_FITC within the reaction. Working bead solutions were initially prepared by diluting AlphaScreen Streptavidin Donor beads to 160 µg/mL and AlphaLISA anti-FITC Acceptor beads to 40 µg/mL in TKMB buffer. Target DNA (*C. coli glyA* 320–514) and the off-target DNA control (*C. jejuni glyA* 320–514) were diluted to 1.0 nM. Oligonucleotide probes Campy_Bio and C. coli_FITC were diluted according to a predetermined concentration series to achieve the final concentrations being tested. For each reaction, 5 µL of both the diluted Campy_Bio and C. coli_FITC were added, along with 2.5 µL of the appropriate DNA fragment (target, off-target, or no DNA control containing sterile water), resulting in a 12.5 µL pre-bead reaction volume. Plates were sealed, briefly centrifuged, and incubated at 50 °C for 30 min. Following this step, 6.25 µL of AlphaScreen Streptavidin Donor beads and 6.25 µL of AlphaLISA anti-FITC Acceptor beads were added, producing a 25 µL total reaction volume. Plates were again sealed, incubated at 50 °C for 30 min, and assayed for Eu as stated under the Oligo-Plex assay format. Final assay concentrations of the oligos being tested ranged from 3.5 to 5 nM for Campy_Bio and from 0.5 to 2 nM for C. coli_FITC. Perkin Elmer’s TrueHits beads (Cat #AP900TB-D/M) served as a positive control during assay development and optimization. However, their values are not reported here since they were not incorporated into the final assay design. Data is the average of at least four independent replicate trials containing no less than three technical replicates per trial.

This procedure was repeated for the detection of *C. jejuni*, except C. jejuni_DIG was used in conjunction with AlphaPlex 545 anti-DIG Tb Acceptor beads at 40 µg/mL in TKMB buffer. Because of its universal nature, the Campy_Bio oligo and the AlphaScreen Streptavidin Donor beads at 160 µg/mL were still employed as above when detecting *C. jejuni*. However, *C. jejuni glyA* 320–514 became the target DNA and *C. coli glyA* 320–514 the off-target DNA control for these assays, with concentrations of 10 nM being added per well. Final assay concentrations of the oligos being tested ranged from 3 to 5 nM for Campy_Bio and from 1 to 3 nM for C. jejuni_DIG, with the assay analyzed using the Tb channel.

### 2.4. Optimization of Temperature for Binding Campylobacter spp.

Assay sensitivity and specificity were evaluated under incubation temperatures ranging from 50 °C to 54 °C using the oligo-Plex system. For *C. jejuni* detection assays, working bead solutions were prepared by diluting AlphaScreen Streptavidin Donor beads to 160 µg/mL and AlphaLISA anti-DIG Acceptor beads to 40 µg/mL in TKMB buffer. The DNA target (*C. jejuni glyA* 320–514) and the DNA off-target control (*C. coli glyA* 320–514) were diluted to 10 nM. Oligonucleotide probes (Campy_Bio and C. jejuni_DIG) were diluted to 25 nM and 7.5 nM, respectively. For each reaction, 5 µL of both the diluted Campy_Bio and C. jejuni_DIG oligos, along with 2.5 µL of the appropriate DNA fragment (target, off-target, or no-DNA control containing sterile water), were added to wells for a 12.5 µL pre-bead reaction volume. Plates were sealed, briefly centrifuged, and incubated at the indicated temperature (50–54 °C) for 30 min. Following this step, 6.25 µL of AlphaScreen Streptavidin Donor beads and 6.25 µL of AlphaLISA anti-DIG Acceptor beads were added for a 25 µL total post-bead reaction volume. Plates were again sealed, incubated at the indicated temperature for 30 min, and assayed for Tb as stated under the Oligo-Plex assay format. The full procedure was repeated with each modified incubation temperature (52–54 °C) for the oligo hybridization and post-bead incubation steps to assess temperature-dependent assay performance.

### 2.5. Oligo-Plex Detection and Differentiation of C. coli and C. jejuni

Following the general protocol listed above for the oligo-Plex assay format, multiplexing oligonucleotide hybridization assays were performed to compare sensitivities using two labeling configurations. The first configuration contained 2.5 µL of a 17.5 nM oligo solution of C. coli_FITC and 2.5 µL of a 15 nM oligo solution of C. jejuni_DIG. By contrast, the second configuration contained 2.5 µL of a 17.5 nM oligo solution of C. coli_DIG and 2.5 µL of a 15 nM oligo solution of C. jejuni_FITC. The remainder of the assay was identical for both configurations, with 5 µL of a 25 nM solution of Campy_Bio and 1.25 µL of a 10 mM solution of ssDNA from either *C. jejuni* (*C. jejuni glyA* 320–514) or *C. coli* (*C. coli glyA* 320–514), along with 1.25 µL of TKMB being added. Mixed DNA reactions contained 1.25 µL of both the 10 mM *C. jejuni glyA* 320–514 and *C. coli glyA* 320–514, with the additional TKMB eliminated so that the volume of all wells remained consistently at 12.5 µL. The no DNA control contained 2.5 µL of TKMB buffer only. After a 30 min incubation in the dark at 54 °C, 12.5 µL of the bead mixture (3.125 µL of the 80 µg/mL AlphaLISA anti-FITC Acceptor beads, 3.125 µL of the 80 µg/mL AlphaLISA anti-DIG Acceptor beads, and 6.25 µL of the 160 µg/mL Alphascreen Streptavidin Donor beads) was added to each well. Plates were sealed and analyzed via a PerkinElmer EnVision plate reader using the AlphaPlex detection program for both Tb and Eu, as stated above. Data is the average of three independent replicate trials containing three technical replicates per trial.

### 2.6. Detection of C. jejuni in Milk

The oligo-Plex was tested in non-fat milk prepared from Signature Select brand milk powder purchased at a local grocer and made according to the manufacturer’s instructions, with 1.6 g of milk powder being reconstituted in 11.25 mL of water. The oligo-Plex configuration for assay 2 (Figure 1D) from above was utilized. Briefly, 2.5 µL of C. coli_DIG (17.5 nM), 2.5 µL of C. jejuni_FITC (15 nM), and 5 µL of Campy_Bio (25 nM) were added to the wells. Then, 1.25 µL of DNA from *C. jejuni* (concentrations ranged from 100 nM to 1 nM, with the dilutions performed in the milk) and 1.25 µL of TKMB were added prior to incubation at 54 °C for 30 min in the dark. Post-incubation, 6.25 µL of the Alphascreen Streptavidin Donor beads (160 µg/mL) and 3.125 µL of both the anti-FITC Acceptor beads (80 µg/mL) and anti-DIG Acceptor beads (80 µg/mL) were added per well. Plates were once again incubated at 54 °C for 30 min in the dark prior to being analyzed on a PerkinElmer EnVision Multilabel Plate Reader using the AlphaPlex Tb/Eu program. Data is the average of two independent replicate trials containing at least three technical replicates per trial.

### 2.7. Statistical Analysis

Data analysis, including graph generation and statistical evaluation, was conducted using JMP version 16.2.0. Group comparisons were performed using Analysis of Variance (ANOVA), and pairwise differences among groups were assessed using Tukey’s Honestly Significant Difference (HSD) test. A significance threshold of 0.05 was applied to all statistical tests performed. Thresholds were determined using decision tree analysis in JMP version 16.2.0, which identified data-driven cutoff values for discriminating target from non-target signals in multiplex assays.

## 3. Results and Discussion

### 3.1. Multiplexing via the Oligo-Plex

Multiplexing assays are highly desirable because they can expand an assay’s power and efficiency while reducing the associated costs. Presently, there are no published reports of a multiplexing assay for the detection of DNA that takes advantage of the “no wash” AlphaPlex technology. Therefore, a novel assay was designed for the detection and differentiation of *C. coli* and *C. jejuni*. This new design builds upon elements of our previously reported oligo-Alpha [18] while greatly expanding its capabilities by enabling the simultaneous discrimination of multiple pathogens. In general, it is a bead-based, chemiluminescent assay whose core technology relies on the proximity-dependent energy transfer from donor to acceptor beads, which is mediated by singlet oxygen. Singlet oxygen is a highly reactive species with a short lifetime (~4 µs), which limits its diffusion radius (~200 nm) and ensures that energy transfer only occurs when acceptor beads are in close proximity to donor beads. Oligos, which contain specific modifications to support their attachment to the donor/acceptor beads, act to bring the beads into proximity by binding to sites located in tandem along a corresponding DNA target (Figure 1). The resulting chemical reaction cascade occurring between the donor and acceptor beads culminates in the emission of light at a wavelength that is unique for each acceptor bead type. This wavelength specificity enables multiplexing, since the different acceptor beads can be distinguished by their emission spectra. Importantly, the spatial confinement of the singlet oxygen minimizes the background signal and cross-talk between channels to enhance the sensitivity and specificity of the assay. Because the entire system operates without direct optical excitation of acceptor beads and relies solely on chemical energy transfer, photobleaching is reduced, and signal stability is improved. This mechanism allows the oligo-Plex presented here to achieve high sensitivity in detecting low-abundance targets while supporting the simultaneous detection of multiple analytes within a single well.

### 3.2. Oligo Design and Constraints

All oligonucleotides used in the assay were designed to target areas within the *glyA* gene. Oligos specific for either *C. coli* or *C. jejuni* were made to allow for differentiation of the two *Campylobacter* species (Figure 1A). Oligos specific to the target were modified with either fluorescein isothiocyanate (FITC) or digoxigen (DIG) to allow conjugation to either the anti-FITC europium acceptor bead or the anti-DIG terbium acceptor bead, respectively. These oligos were paired with a universal oligo that resided upstream and contained a dual biotin modification for conjugation to the streptavidin-coated donor beads in the assay. Unlike oligos designed for PCR-based applications, oligos in an oligo-Alpha are all made to the same strand, which, in this case, the antisense strand was arbitrarily chosen. Because oligos anneal to single-stranded DNA, it is important that both donor and acceptor oligo/bead complexes bind to the same strand for efficient energy transfer between the beads, regardless of whether the sense or antisense strand is chosen as the target.

Since Alpha-based technologies rely on the transfer of energy from donor beads to acceptor beads through singlet oxygen molecules, the diffusion radius of ~200 nm for a singlet oxygen molecule is a critical parameter around which the assay must be designed. Therefore, based upon calculations using 0.6 nm as the distance between base pairs in ssDNA and 0.34 nm for dsDNA [23], all acceptor oligos should reside within 333–588 bp of the donor oligo, respectively. In this study, the acceptor for *C. coli* was located further from the donor compared to that for *C. jejuni*. Regardless, both were well within the maximum allowable distance.

Oligos were evaluated using BLASTn (https://blast.ncbi.nlm.nih.gov/Blast.cgi?PROGRAM=blastn&PAGE_TYPE=BlastSearch&LINK_LOC=blasthome (accessed on 18 August 2026) with the parameters adjusted for short input sequences (National Center for Biotechnology Information) to help identify partial homology, low-stringency binding sites, and cross-species conservation. This in silico analysis found one exact match to C. jejuin_DIG in an alternative *Campylobacter* species (*C. molothri*). Not only is this species associated with non-domesticated birds [24] but it also contains a single nucleotide polymorphism (SNP) within the Campy_Bio binding site. Given that previous research has shown that oligos incorporating a SNP can differentiate targets [18], it is unclear if signal generation would occur with *C. molothri*. Conversely, C. coli_DIG was not identical to any sequences within the database. The closest alignments showed only partial identity (20 out of 23 nucleotides with *Spirochaetia* bacterium and 19 out of 23 nucleotides for *Corynebacterium pseudotuberculosis*). Not unexpectedly, several organisms displayed perfect identity to Campy_Bio, including *Clostridium saccharoperbutylacetonicum* and *C. septicum*. However, these organisms lack the required downstream binding regions for the second oligonucleotides, thus preventing successful signal transfer if they were present within the sample.

### 3.3. Optimization of Oligo Concentration and Temperature

**Figure 2:**
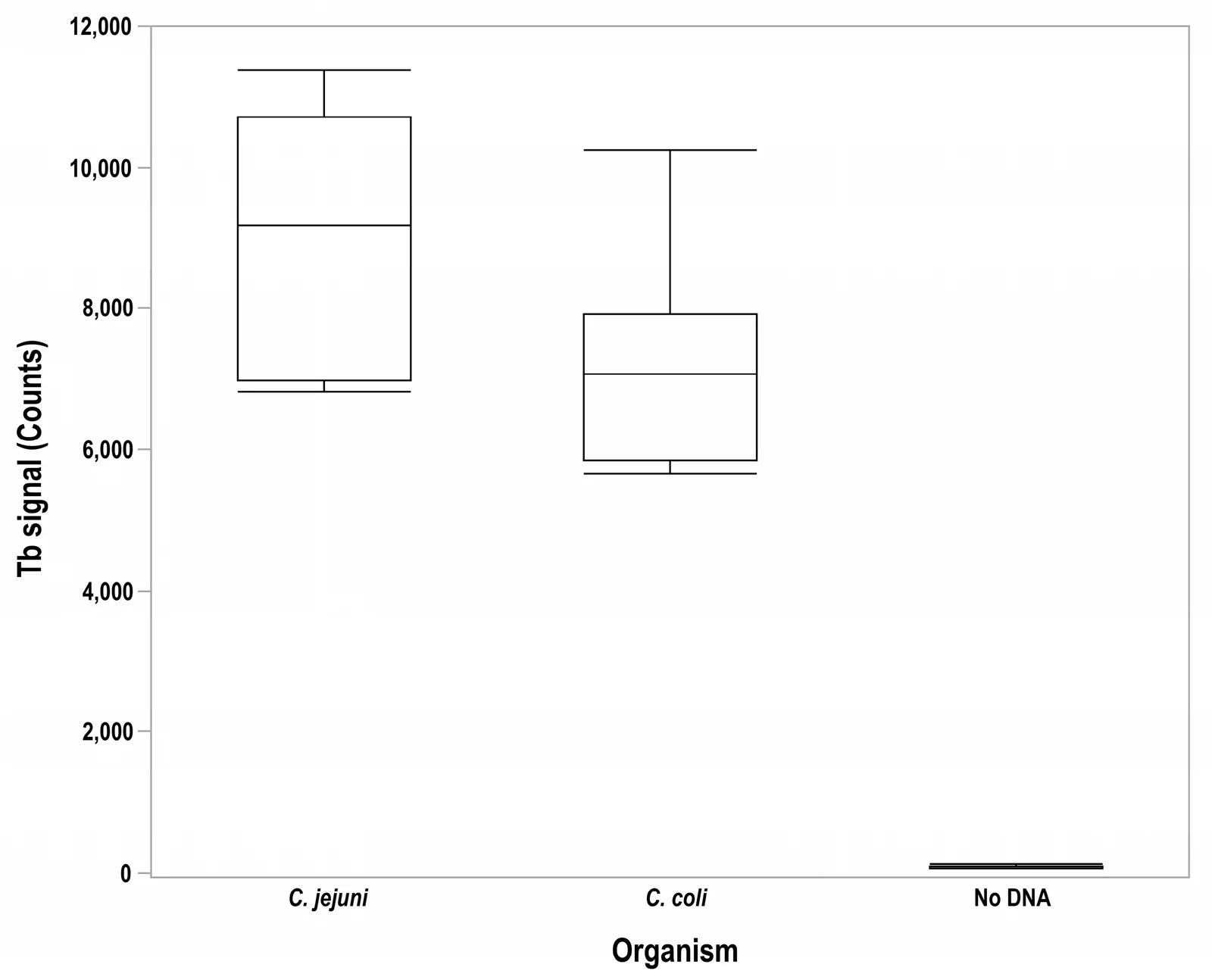
Signal overlap for *C. coli* and *C. jejuni* at 50 °C. Overlapping signal intensity prevents the reliable discrimination of *C. jejuni* (target) from *C. coli* (non-target) when assayed at 50 °C. Only the no DNA control was clearly distinguishable from both target and non-target at this temperature.

Optimal concentrations of the donor and acceptor oligos were determined empirically by varying each within the assay (Supplemental Figure S2). For the detection of *C. coli*, signal intensities were highest using 4.5 nM of the Campy_Bio oligo and 1.75 nM of C. coli_FITC, with a mean signal of ~173,713 units in the Eu channel. Although the concentrations were relatively similar, signal intensities for the detection of *C. jejuni* peaked at a concentration of 5 nM of the Campy_Bio oligo and 1.5 nM of the C. jejuni_DIG oligo, with a mean signal of ~6310 units in the Tb channel. Despite optimization of the oligonucleotide concentration, a combination of high background and lower overall signal intensity impeded the ability to reliably distinguish *C. jejuni* from *C. coli* at 50 °C using the Tb channel (Figure 2). To help enhance oligo specificity and decrease background signals, hybridization temperatures ranging from 50 to 54 °C were evaluated.

**Figure 3:**
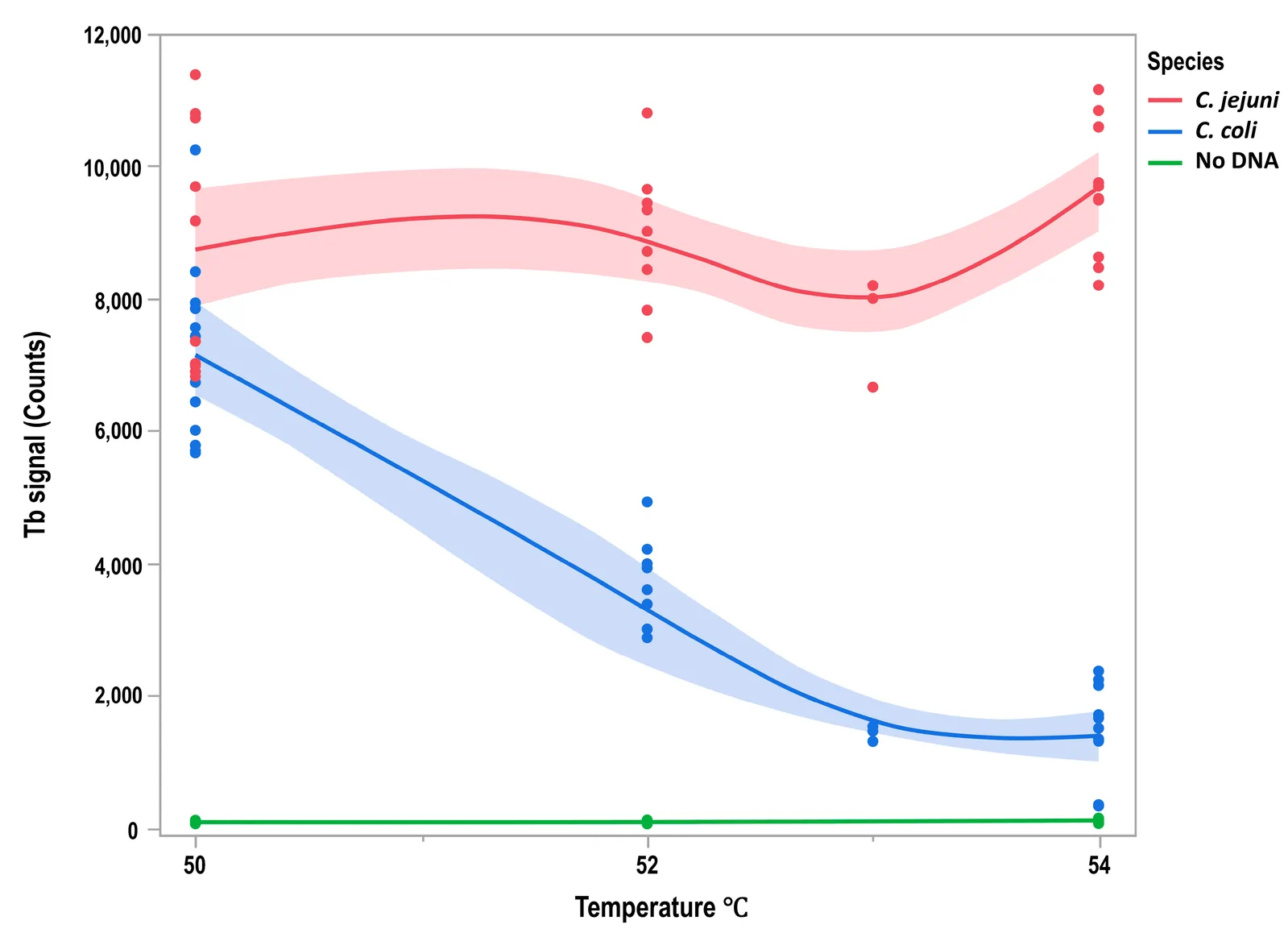
Effect of temperature on assay signal for target and non-target species. Signals obtained from the *C. jejuni* target (red) and the *C. coli* non-target (blue) were altered as the reaction temperature was varied (50–54 °C). The no DNA control (green) remained relatively unchanged across the same temperature span. Individual samples, measurement averages, and standard deviations are noted by colored dots, lines, and bands, respectively.

In general, temperature changes showed a positive correlation with target signals and a negative correlation with signals from non-target organisms. This ultimately increased the signal-to-noise ratio so that there was a clear separation between *C. jejuni* and *C. coli* at 54 °C using the Tb beads (Figure 3). Temperature adjustments provide a clean, reliable, and controllable means by which hybridization specificity in the oligo-Alpha could be increased. It selectively destabilizes mismatched duplexes without altering bead chemistry, generating background artifacts, or interfering with lanthanide-based signal generation. It also does not increase the cost or complexity of the assay. By contrast, chemical additives can be used for destabilization but may introduce undesirable effects such as altering antibody affinities, bead surface chemistries, the lifespan of the oxygen radical, and donor/acceptor optical behavior, all of which can reduce assay reproducibility. Therefore, temperature should be used preferentially over chemical methods to increase stringency when designing oligo-Alphas.

### 3.4. Signal Intensity and Differentiation of C. coli and C. jejuni Using the Oligo-Plex

Several factors are known to affect signal intensity. For example, bead choice influences the signals obtained, as europium beads typically generate stronger and more reliable alpha signals than terbium beads. The narrow emission and long luminescent lifetime of europium help promote low background interference and improve signal-to-noise performance. Although terbium exhibits a higher quantum yield, its emission overlaps more with biological autofluorescence [25], making europium the superior choice. In addition, the FITC label is smaller and more accessible than DIG, which reduces steric constraints and enhances antibody recognition on bead surfaces. Anti-FITC reagents also tend to display a more consistent affinity and have been optimized for bead conjugation because of their widespread use, thus further supporting effective donor/acceptor bead interactions. Together, these factors make the FITC–europium system inherently more favorable for oligo-based Alpha assay configurations than the DIG–terbium counterpart.

**Figure 4:**
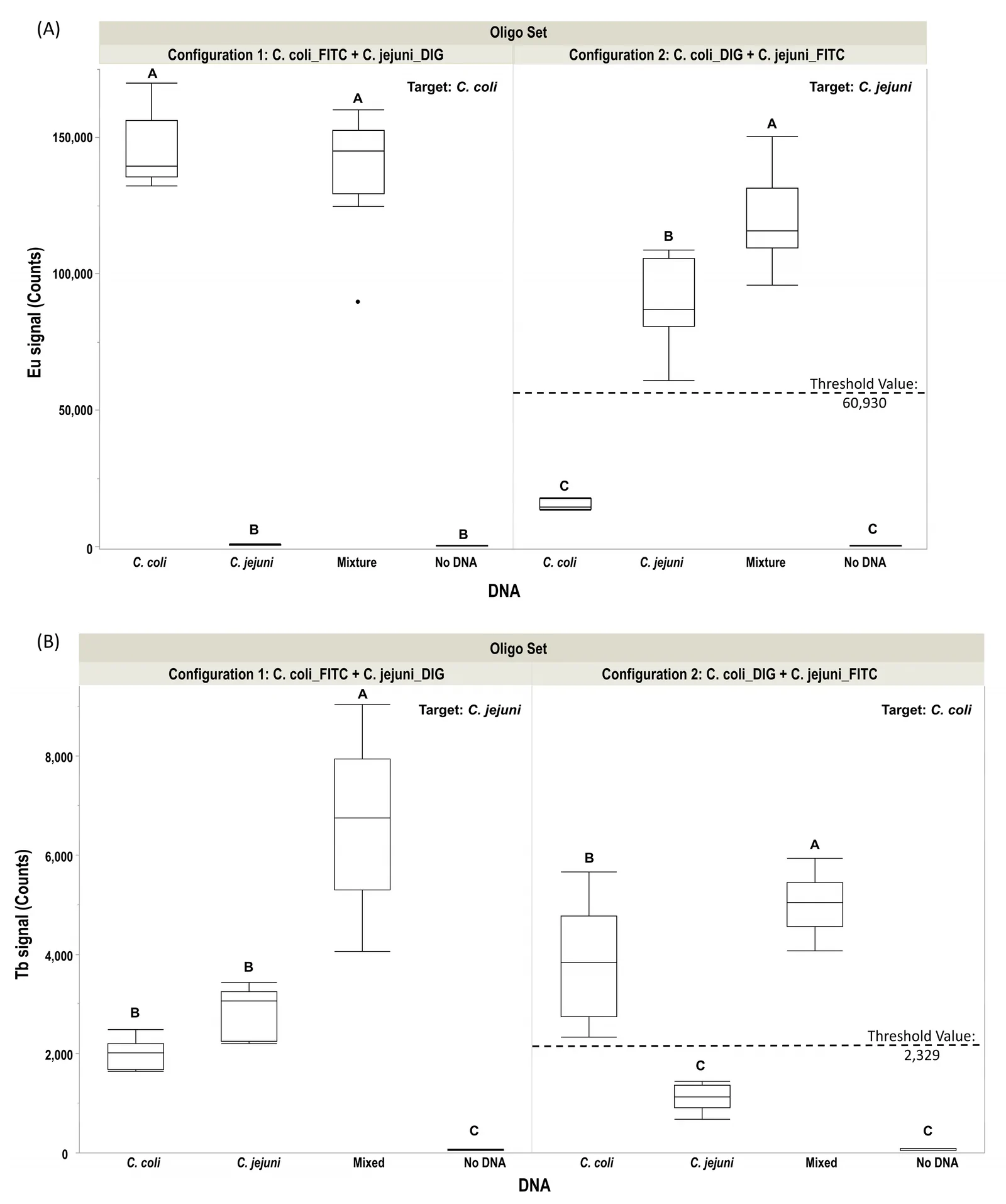
Oligo-Plex for the differentiation of *Campylobacter*. Multiplex detection of *C. coli* and *C. jejuni* was conducted using two assay configurations (bead configuration 1 (left) and bead configuration 2 (right)). For each configuration, signals obtained in the (**A**) europium and (**B**) terbium channels were recorded. Putative threshold values are denoted by dotted lines. Groups sharing the same letter are not significantly different, as determined by the Tukey–Kramer HSD test.

To assess the effect of these factors on target detection in an oligo-Plex format, the oligo-Plex assay was employed for the multiplex detection of *C. coli* and *C. jejuni* using the optimized oligo concentrations (5 nM of the universal Campy_Bio oligo, 1.75 nM of the *C. coli*-specific oligo, and 1.75 nM of the *C. jejuni* specific oligo) and temperature (54 °C) identified. It is worth noting that the higher concentration of Campy_Bio (5.0 nM versus 4.5 nM) from the previous testing was employed in the oligo-Plex since this amount provided the strongest signal under the weakest assay conditions (i.e., terbium beads). Testing was conducted using two detection configurations (Figure 4). In the first, *C. coli* was detected with europium-acceptor beads and *C. jejuni* with terbium-acceptor beads (Figure 1D-Assay 1). In the second configuration, this was reversed, with *C. coli* being detected using terbium-acceptor beads and *C. jejuni* detected using europium-acceptor beads (Figure 1D-Assay 2). As expected, the europium acceptor beads consistently produced higher signal values regardless of whether the target was *C. coli* or *C. jejuni*. A clear separation between target and non-target was observed with both configurations when using the anti-FITC europium acceptor beads (Figure 4A). However, when the anti-DIG terbium acceptor beads were used to detect *C. jejuni*, a clear separation was not achieved with one of the two configurations. In this assay, the non-target *C. coli* signal overlapped with the target *C. jejuni* signal (Figure 4B). This is in contrast to having the anti-DIG terbium acceptor beads detect *C. coli*, where a clear separation was observable, thus allowing for the establishment of threshold values for all target organisms. Consequently, design of the oligo-Plex requires the employment of the anti-DIG terbium acceptor beads for *C. coli* detection and the anti-FITC europium acceptor beads for detection of *C. jejuni*.

Interestingly, in these assays, higher signals were consistently obtained for *C. coli* over *C. jejuni* irrespective of the labeling system used (Figure 4) and despite the acceptor binding to a site a greater number of nucleotides away from the donor along the target DNA strand (Figure 1A). For example, the average signal was 144,900 ± 12,810 (Supplemental Table S2) when *C. coli* was labeled with FITC and read on the Eu channel, whereas it was only 89,654 ± 15,462 when *C. jejuni* was labeled with FITC and read on the same channel. Similarly, the average signal was 5807 ± 2926 when *C. coli* was labeled with DIG and read on the Tb channel, compared to 2675 ± 585 for *C. jejuni* using the same assay configuration. Based on these results, additional factors are likely influencing the signal generated by a set of donor/acceptors within these oligo-based assays. For instance, the spatial arrangement within the *C. coli* assay may be more favorable due to the positioning of the donor relative to the acceptor bead within the context of the double helix. Given that the DNA helix contains 10.4 base pairs per turn in solution [26], in the *C. jejuni* assay, the acceptor bead is ~8.0 turns away from the donor, which places both beads along the same face of the helix. For the *C. coli* assay, the acceptor bead would be located ~13.5 turns away from the donor, indicating the beads sit on opposite faces of the helical strand. This configuration may be more conducive to binding, since it would likely minimize steric hinderance between the two beads. Although not tested here, the addition of linkers between the oligo and the attached tag may also aid in obtaining higher signals by allowing more flexibility in the angular orientation of the beads and reducing hybridization interference.

### 3.5. Buffer Compatibility

The oligo-Plex assay uses oligos labeled with biotin, FITC, and DIG, allowing for attachment to streptavidin, anti-FITC, and anti-DIG-coated beads. This is significant because it permits amine-based buffers to be employed in the reaction since binding relies exclusively on non-covalent molecular interactions. Unlike traditional AlphaLISA bead conjugation that uses amine-reactive chemistries (like NHS-ester activation) to covalently attach antibodies to beads, free amines in the buffer do not compete or interfere with binding. Therefore, Tris, which is commonly found in DNA-based detection assays, can be utilized. TKMB buffer was chosen because it stabilizes nucleic acids by (1) efficiently buffering the pH in the physiological range, (2) protecting against acid-catalyzed depurination, and (3) helping to maintain the electrostatic environment required for stable DNA hybridization. Because Tris-based buffers do not disrupt hydrogen bonding/base stacking within duplex DNA or interfere with non-covalent affinity tags, the protocol is simplified as oligos can be prepared, hybridized, and used directly in the oligo-Plex without the need for a tedious buffer exchange.

### 3.6. Oligo-Plex Detection of C. jejuni in Milk

**Figure 5:**
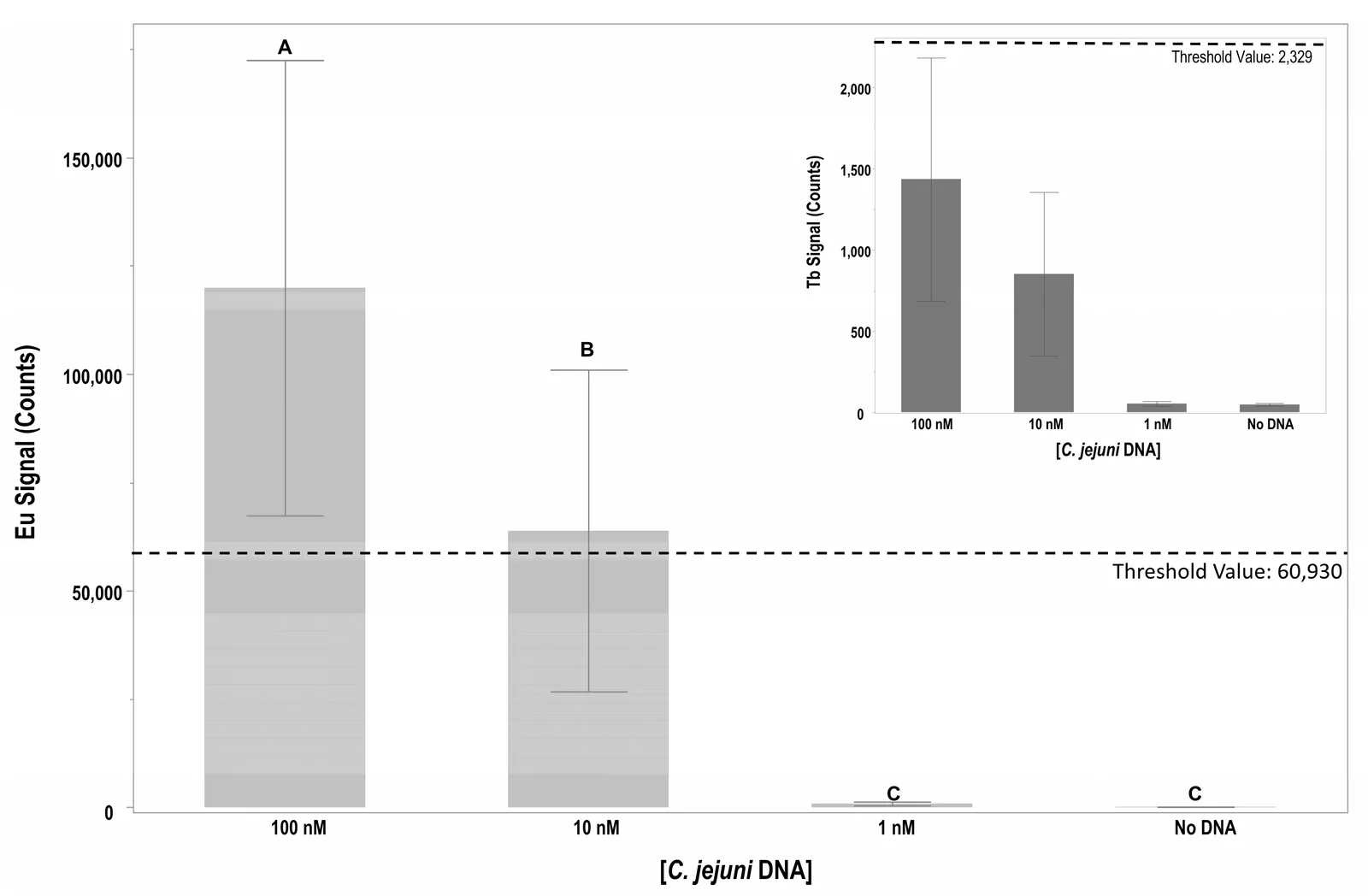
Detection of *C. jejuni* in milk via the oligo-Plex. The oligo-Plex was evaluated for its ability to detect *C. jejuni* in non-fat milk inoculated with a serial dilution of *C. jejuni* DNA (100 nM, 10 nM, and 1 nM). Signals generated from the europium channel from the oligo-Plex assays containing ≥10 nM of target DNA were above the threshold level set for the assay, demonstrating the ability to detect *C. jejuni* in milk. All signals from the terbium channel (inset) were below the threshold level, demonstrating the absence of *C. coli*. Mean signal values are shown with error bars representing the standard deviation. Groups sharing the same letter are not significantly different, as determined by the Tukey–Kramer HSD test.

Although the assay was originally intended to detect purified DNA in TKMB, this oligo-Plex configuration was also evaluated for its compatibility with an alternative sample matrix. Milk was selected as DNA from dead cells could be present in this matrix based upon reports of *Campylobacter* contamination and its persistence in raw milk products [27,28]. Due to accessibility, the oligo-Plex was not tested in raw milk but was instead conducted in non-fat milk spiked using various levels of *C. jejuni* DNA fragments. These assays demonstrated the ability of the oligo-Plex to successfully detect the *C. jejuni* target. Signals produced in the europium channel at the 100 nM and 10 nM DNA concentrations were above the putative threshold level set for the assay (Figure 5), indicating the presence of *C. jejuni*, whereas none of the signals produced in the terbium channel were above the threshold level (Figure 5 inset), indicating the absence of *C. coli*. It is also worth noting that the average signal for samples containing 1 nM of *C. jejuni* DNA was substantially higher (904 ± 438) than the no DNA control (154 ± 25), although the difference was not statistically significant. This may be due in part to the larger signal variances seen at the higher DNA concentrations. Nevertheless, because the thermostability of the oligo-DNA duplexes may differ between TKMB buffer and milk, temperature adjustments or other modifications may be possible to further optimize the assay’s performance and achieve a lower limit of detection.

### 3.7. Technical Challenges and Limitations

Although the use of alternative matrices is possible, some matrices, such as raw milk or other food products, could result in decreased signal intensities due to elevated levels of background microorganisms and/or additional organic debris that could interfere with the assay components. This susceptibility to background signal issues can complicate assay reliability. It also requires empirical evaluations to be performed with each matrix until a more general understanding of specific factors that cause interference can be defined. In addition, the need for multiple modified oligonucleotides, specialized beads, and a plate reader capable of performing Alpha-based detection increases the overall cost of the assay, even though its high throughput is advantageous.

Several limitations inherent to the assay should also be noted. For example, its performance is still dependent upon oligo design, bead chemistry, and uninhibited singlet oxygen energy transfer. As demonstrated here, signals can vary substantially across bead types, with terbium-based detection consistently underperforming relative to europium. Moreover, with only two bead chemistries (terbium and europium) currently commercially available, multiplexing capabilities are severely limited compared to PCR-based assays using fluorescent probes. The oligo-Plex also shows signs of steric constraints related to bead orientation and/or tag accessibility. This confines target selection to genomic regions where spacing is favorable, which limits assay configurations and reduces flexibility. Additionally, because the method relies on hybridization rather than enzymatic amplification, its fundamental detection limit is higher than that of PCR or other techniques utilizing nucleic acid amplification. Further testing is needed to establish the analytical sensitivity of the oligo-Plex relative to techniques such as PCR. Collectively, these limitations highlight that while the oligo-Plex described here offers a unique multiplexing technique in a no-wash ELISA format, additional research is required before it can be reliably applied to field-derived samples.

## 4. Conclusions

As the first demonstration of an AlphaPlex/oligo-Alpha configuration for the differentiation of multiple nucleic acid-based targets, this method establishes a streamlined and adaptable framework for nucleic acid-based detection of various pathogens. Because the oligo-Plex platform relies on proximity-based bead signaling and simple reagent addition steps, it is well suited for high-throughput testing where speed, sensitivity, and automation are essential. Incorporating oligonucleotide probes in place of antibodies allows highly specific detection and can make it applicable where traditional immunological assays may fail. By screening for multiple targets at once, it can lend insight into products with multiple contamination events while greatly reducing sample loads and time-to-results. This ultimately extends the versatility of the oligo-Alpha system, allowing it to be adapted to a broad range of pathogens beyond *Campylobacter* through the selection and implementation of appropriate oligos.

## Data Availability

The original contributions presented in this study are included in the article/Supplementary Material. Further inquiries can be directed to the corresponding author.

## Supplementary Materials

The following supporting information can be downloaded at https://www.mdpi.com/article/10.3390/pathogens15090884/s1; Figure S1: Workflow for the oligo-Plex. Schematic of the processes and procedures for the oligo-Plex. Total workflow time ~75 min. Figure S2: Oligo optimization for *Campylobacter* assay. Optimal oligo concentrations were determined for both the (**A**) *C. coli* and (**B**) *C. jejuni* assays by varying the concentration of the Campy_Bio oligo in addition to either the C. coli_DIG or C. jejuni_DIG oligos. Optimal concentrations were defined by values yielding the highest signal with the lowest amount of variation. Table S1: Primers and single-stranded DNA fragments used for the oligo-Plex. All sequences are representative of *Campylobacter*’s *glyA* gene. Table S2: Signal means obtained from the Europium and Terbium channels when using ssDNA of *C. coli*, *C. jejuni*, a mixture of *C. coli* and *C. jejuni*, or no DNA.

## Abbreviations

The following abbreviations are used in this manuscript:

ELISA	Enzyme-linked immunosorbent assays
AlphaLISA	Amplified luminescent proximity homogenous assay-linked immunosorbent assay
