# Supplementary material for: Novel AlphaPlex Design Enables Rapid Differentiation of Campylobacter Species
Source: Pathogens. 2026 Aug 24;15(9):884. doi: 10.3390/pathogens15090884 (PMC13609963; doi:10.3390/pathogens15090884)
Supplement: Supplementary file 1 [file pathogens-15-00884-s001.zip › Supplementary Tables S1 and S2.pdf]

Supplemental Table S1: **Primers and single stranded DNA fragments used for the oligo-Plex. All sequences are representative of *Campylobacter*'s *glyA* gene.**

Target and primer/oligo name	Sequence	Location within *glyA*	Oligo Modification	Reference
**Donor bead oligo**				
Campy_Bio	CACCATGACTTAAATCCATTCC	359-337	5' biotin	This study
**Acceptor bead oligos**				
C. jejuni_DIG	TCTTCCATCAAGTTCTACGCC	441-420	5' DIG	This study
C. jejuni_FITC	TCTTCCATCAAGTTCTACGCC	441-420	5' FITC	This study
C. coli_FITC	CACGATAAGCTTTGGTTTTACAA	498-476	5' FITC	Modified from [22]
C. coli_DIG	CACGATAAGCTTTGGTTTTACAA	498-476	5' DIG	Modified from [22]
***glyA* target DNA**				
C. coli_glyA	CAGGTGATAAAATTTTAGGAATG GATTTAAGTCATGGTGGACATTT AACACATGGTGCAAAAGTAAGC TCATCGGGAAAAATGTATGAGA GCTTTTTTTATGGTGTAGAGCTT GATGGAAGGATAAACTATGAAA AAGTTAGGGAAATTGCTCATAT TGTAAAACCAAAGCTTATCGTG TGCGGTGCGAGTGCTT	320-514	-	GenBank ID CP015528.1
C. jejuni _glyA	CAGGTGATAAAATTTTAGGAAT GGATTTAAGTCATGGTGGACATT TAACTCATGGTGCAAAAGTAAG TTCTTCGGGTAAAATGTACGAAA GTTGTTTTTACGGCGTAGAACTT GATGGAAGAATTGATTATGAAA AAGTAAGAGAAATTGCTAAAAA AGAAAAGCCAAAATTAATAGTT TGTGGAGCTAGTGCTT	320-514	-	GenBank ID CP041584.1

Supplemental Table S2: **Signal means obtained from the Europium and Terbium channels when using ssDNA of *C. coli, C. jejuni,* a mixture of *C. coli* and *C. jejuni,* or no DNA.**

Europium Channel				
	C. coli	*C. jejuni*	*C.coli+C. jejuni*	no DNA control
C. coli_FITC+ C. jejuni_DIG	144900±12810	8001±150	138433±21174	471±54

C. jejuni_FITC+ C. coli_DIG	15281±1888	89654±15462	119578±17237	369±112

Terbium Channel				
	C. coli	*C. jejuni*	*C.coli+C. jejuni*	no DNA control
C. coli_FITC+ C. jejuni_DIG	3751±2591	2675±585	5125±1748	751±1203
C. jejuni_FITC+ C. coli_DIG	5807±2926	1238±267	4057±1643	449±693
